# Antipsychotic Misuse: A Silent but Growing Public Health Hazard

**DOI:** 10.7759/cureus.98275

**Published:** 2025-12-01

**Authors:** Sumeet Bhardwaj, Karisma Pathak, Myra McLenon, Steven Spector, Kevin Tu

**Affiliations:** 1 Psychiatry, Kansas City University, Kansas City, USA; 2 General Medicine, Kansas City University, Kansas City, USA; 3 General Medicine, Kansas City University of Medicine and Biosciences, Kansas City, USA

**Keywords:** antipsychotic drugs, cardiometabolic risk, cardiovascular disease, health disparities, insulin resistance, metabolic monitoring, metabolic syndrome, off-label prescribing, pharmacovigilance, second-generation antipsychotics

## Abstract

Antipsychotic drugs are essential in the management of schizophrenia, bipolar disorder, and other psychiatric illnesses, but their use is closely linked to cardiometabolic side effects. Cardiometabolic side effects include weight gain, dyslipidemia, glucose intolerance, and increased cardiovascular morbidity and mortality. Mechanisms of cardiometabolic side effects involve disruption of the central nervous system’s ability to regulate appetite, peripheral metabolic effects, which are changes in metabolism outside the CNS, and receptor-level interactions, with second-generation antipsychotics generally posing a greater risk than first-generation agents. This review addresses current evidence on the cardiometabolic consequences of antipsychotic therapy, increasing inappropriate off-label use of antipsychotic drugs, particularly those that are atypical, highlights vulnerable populations, and discusses strategies for monitoring and mitigation. A secondary goal is to address the considerable literature gap covering recent trends and costs in the off-label use of atypical antipsychotic drugs.

## Introduction and background

Antipsychotic drugs have transformed psychiatric care by enabling effective management of psychotic symptoms and allowing millions of individuals with severe mental illness to live outside institutional settings and to be spared treatments that would be considered to be outdated today [1]. Beyond schizophrenia, antipsychotics are also prescribed for bipolar disorder, treatment-resistant depression, and a variety of pediatric and developmental disorders [2]. This review does not aim to portray antipsychotics as inherently harmful, but rather to highlight that their expanding use demands careful risk-benefit assessment, particularly regarding atypical antipsychotics and their potential cardiometabolic effects.

Although antipsychotics have transformed psychiatric care, their use has also extended far beyond approved indications. In this review, off-label refers to the prescription of antipsychotic medications for conditions outside their original regulatory approval. While a handful of off-label uses, such as for treatment of major depressive disorder, augmentation in treatment-resistant obsessive-compulsive disorder, or management of severe agitation, have modest empirical support, most other applications lack any convincing evidence.

Despite their widespread use for insomnia, anxiety, personality disorders, dementia-related behaviors, and post-traumatic stress disorder, controlled trials generally show minimal or no benefit [3]. In practice, the vast majority of off-label antipsychotic prescribing reflects clinical habits despite a lack of sufficient evidence to alleviate issues such as insomnia and anxiety due to their strong antihistaminergic activity, and it is more common when clinicians perceive a lack of effective alternatives or face therapeutic dead-ends.

The rapid rise in such prescribing has emerged as a significant public-health concern [2]. National data indicate that among atypical antipsychotics, visits involving off-label use with uncertain evidence increased from 0.44 million (45%) in 1995 to 6.9 million (54%) in 2008, a nearly tenfold escalation in utilization [2].

In contrast, typical antipsychotics showed a decrease from 3.6 million visits (76%) to 0.8 million (65%) over the same period. However, this apparent decline can be argued to be potentially deceptive as it may reflect atypical agents increasingly replacing their predecessors across both approved and unapproved indications rather than improved prescribing selectivity.

Further evidence shows that in 1995, 74% of all antipsychotic treatment visits (≈4.4 million) were for non-FDA-approved conditions, compared with 60% (≈9.0 million) in 2008 [2]. While this data can suggest a modest proportional decline, the absolute number of off-label visits more than doubled, signaling an overall expansion in patient exposure [2]. Moreover, it is known that atypical antipsychotics accounted for 50% of off-label use in 1995, rising to 66% in 2003 before leveling at 60% in 2008, whereas typical agents declined from 78% to 67% [2]. Collectively, these findings add more evidence that there is a redistribution of prescribing patterns rather than a genuine reduction in off-label practice. This pattern can be explained by several forces: the widespread perception of atypicals as safer alternatives, aggressive pharmaceutical marketing during the 2000s, and the lack of comparably effective agents for behavioral control.

Prescribing patterns have yielded evidence that off-label atypical antipsychotic drugs are expanding rapidly among children and older adults [2]. The cardiometabolic risks of atypical antipsychotic drugs among these populations are particularly concerning, as children can suffer a lifetime of increased morbidity and mortality from complications of developing diabetes, and the elderly could be put at increased risk of being disabled from strokes due to dyslipidemia. The number of treatment visits among children for antipsychotic drugs increased eightfold from 1995 to 2005 [2]. Unless a true reduction in off-label practices is put into practice, it is more than likely that it will continue unchecked.

Alarmingly, recent evidence strongly suggests that the problem of off-label antipsychotic prescribing has persisted into the modern era largely unresolved. A 2021 retrospective repeated-panel analysis examining monthly off-label utilization of second-generation antipsychotics (SGA) among adults with fee-for-service Medicare, Medicaid, and dually eligible coverage across White, Black, and Latino populations found that comprehensive national data on current trends, dosing patterns, and costs remain largely unavailable [4]. There may also be an unintended economic cost in the off-label prescriptions of atypical antipsychotic drugs, borne in their propensity to increase the risk of developing diabetes and other cardiovascular diseases [4]. The cost is present among African-American and Latino populations, given their higher baseline level of cardiovascular disease than Whites [4].

Evidence shows that SGA, particularly clozapine and olanzapine, confer the highest metabolic liability, producing significant increases in body weight, triglycerides, and insulin resistance within months of initiation [5]. These adverse effects contribute substantially to the two- to three-fold higher cardiovascular mortality observed in patients receiving long-term antipsychotic therapy. These groups are also significantly less likely to receive recommended metabolic monitoring, such as lipid and glucose testing, which may delay recognition and intervention for emerging cardiometabolic disease [6].

African American and Latino racial-ethnic groups tend to have lower socioeconomic status and access to healthcare resources, and thus are suffering disproportionately from increasing antipsychotic utilization. The economic costs have been concluded to have been largely carried by the two main public payers, Medicaid and Medicare [7,8]. Although evidence supporting many off-label indications for antipsychotic medications remains limited, their use should be approached with caution. Potential inefficiencies or increased risk of adverse effects demand consideration; however, it is equally important to acknowledge that off-label prescribing often emerges in situations where conventional treatments have been ineffective, poorly tolerated, or clinically insufficient. As such, characterizing off-label antipsychotic use as definitively “low-value care” is premature without more rigorous data.

A more balanced interpretation recognizes both the potential for harm and the clinical uncertainties that frequently motivate off-label decisions. Within financially constrained public payer systems, off-label utilization may still carry implications for resource allocation, potentially diverting funds from higher-value interventions. Therefore, careful, individualized prescribing supported by stronger evidence is essential to ensure both clinical appropriateness and responsible stewardship of limited healthcare resources.

A substantial proportion of individuals prescribed antipsychotics had no recorded psychiatric diagnosis; in fact, two out of five patients (26%-36%) lacked any documented mental illness [4]. Given the well-known side effects of antipsychotic drugs, this discovery poses deep ethical concerns. Interestingly, there is also no available information on the specialty or discipline of the prescribers, making it difficult to evaluate how provider type may be associated with trends in off-label antipsychotic prescribing. However, recent evidence suggests that although psychiatrists may be more likely to prescribe antipsychotics off-label, nonpsychiatrists appear to be far more likely to prescribe antipsychotics without documenting a mental illness diagnosis [9]. This revelation further supports our previous assertion of ethical concerns as it poses not only a danger to patients, but to the prescribers because of legal and economic consequences.

Nonpsychiatrists may furthermore be unaware that there is a limited amount of evidence on the dose-dependent relationship of cardiometabolic effects of atypical antipsychotics in those with mental illnesses, and consequently, the dosages of atypicals being prescribed may be far less protective than they originally had assumed [10,11]. This may imply either a deficiency in established literature regarding antipsychotics or that their training may be deficient in comparison to psychiatrists in utilizing these agents. The continued absence of detailed, up-to-date surveillance suggests that the extent of inappropriate prescribing may, in fact, be underestimated. These findings reinforce the need for heightened clinical vigilance when initiating antipsychotics for non-approved indications, as their benefits in such contexts are uncertain while their risks, particularly metabolic and neurologic, remain well established.

## Review

Methods

This narrative review was conducted to gather current evidence on the increasing use of antipsychotics for off-label purposes and potentially emerging misuse patterns. Relevant literature was identified primarily through PubMed and Google Scholar, focusing on studies published in peer-reviewed journals. The search prioritized recent reviews, large cohort studies, consensus statements, and meta-analyses examining antipsychotic use, cardiometabolic risk, and off-label prescribing trends.

Additional references were obtained by manually screening the bibliographies of key articles to ensure comprehensive coverage. Eligible studies were included if they discussed the metabolic, cardiovascular, or public-health impacts of antipsychotic therapy. Only English-language, full-text, peer-reviewed publications were considered.

The selected literature was analyzed and synthesized thematically, emphasizing recurring patterns in off-label prescribing, differential risk profiles among antipsychotic generations, and evidence-based strategies for monitoring and mitigation. Because of the diversity in study design and outcome measures, a qualitative synthesis was chosen over quantitative analysis to provide an integrative understanding of the topic.

**Figure 1:**
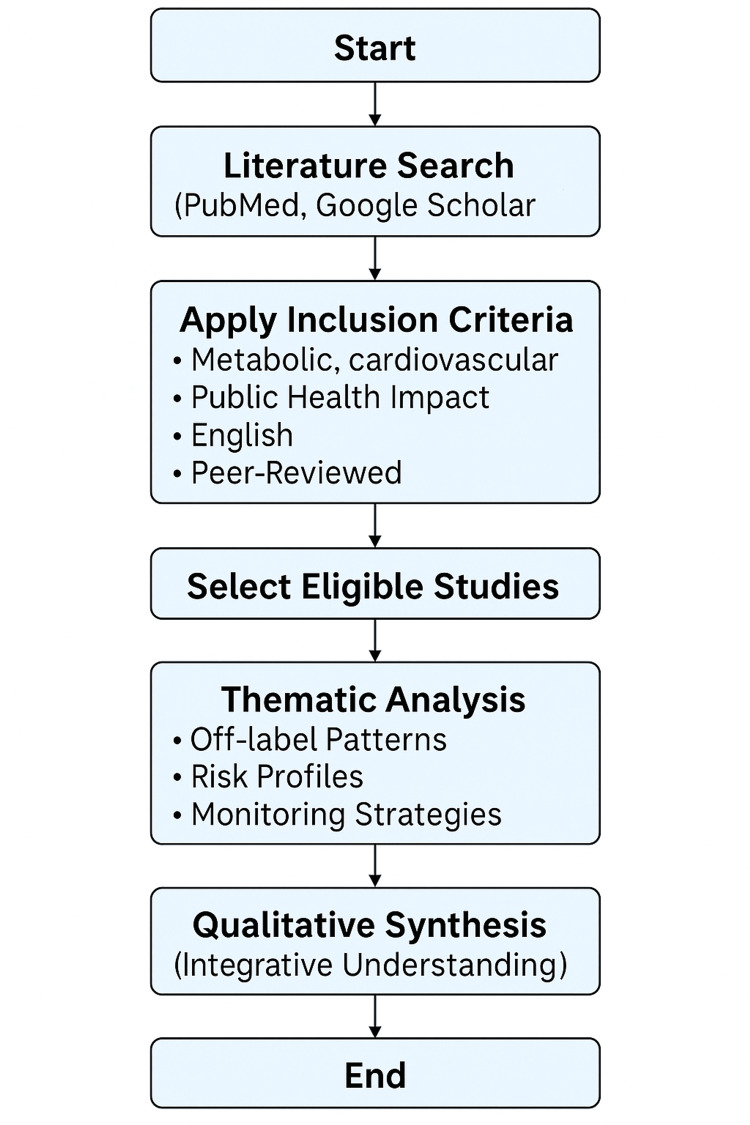
Flow Methodology

Classification of antipsychotics

For the purposes of this review, it is first necessary to define what constitutes an antipsychotic drug and the categories in which they fall. Broadly, there are two generations of antipsychotic drugs, with an emerging third generation. Table 1 will help to provide a visualization of antipsychotic drugs and their key characteristics.

First-generation antipsychotics (FGAs) such as haloperidol, fluphenazine, and chlorpromazine primarily exert their effects through potent antagonism of dopamine D₂ receptors [12]. This blockade makes FGAs highly effective for controlling the positive symptoms of schizophrenia, including hallucinations, delusions, and agitation [12]. However, antagonism in the nigrostriatal pathway produces a high risk of extrapyramidal symptoms (EPS), including acute dystonia, Parkinsonism, akathisia, and tardive dyskinesia, the latter of which can be irreversible [12]. Neuroleptic Malignant Syndrome, although rare, represents the most severe acute complication and can be life-threatening [12]. FGAs are less effective for negative symptoms and cognitive deficits, and their use is often limited by poor tolerability [12]. Metabolic side effects such as dyslipidemia are generally less pronounced than with second-generation agents, though low-potency FGAs like chlorpromazine may still cause weight gain and sedation [12].

SGAs, including clozapine, olanzapine, risperidone, quetiapine, aripiprazole, and lurasidone, were developed to reduce neurological side effects while improving efficacy [12]. SGAs antagonize both dopamine D₂ and serotonin 5-HT₂A receptors; the latter action increases dopamine release in the nigrostriatal pathway and thereby lowers EPS risk [12]. They also interact with histamine, muscarinic, and adrenergic receptors, contributing to sedation, anticholinergic effects, orthostatic hypotension, and metabolic disturbances [12]. Compared with FGAs, SGAs demonstrate improved efficacy in treating negative symptoms and may have advantages in mood stabilization, making them useful in bipolar disorder and as adjuncts in depression [12]. However, these benefits are accompanied by greater cardiometabolic risk, particularly with clozapine and olanzapine, which are strongly associated with weight gain, insulin resistance, dyslipidemia, and diabetes [12]. Other SGAs, such as aripiprazole, lurasidone, and ziprasidone, carry lower metabolic risk, underscoring the importance of individualized selection.

Third-generation antipsychotics (TGAs) have recently emerged and are characterized by partial agonism at dopamine D₂ receptors rather than full antagonism [12]. Agents such as aripiprazole, brexpiprazole, and cariprazine act as “dopamine system stabilizers,” modulating dopaminergic activity upward when it is deficient and downward when excessive [12]. TGAs aim to preserve normal dopaminergic tone while reducing the risk of extrapyramidal symptoms, hyperprolactinemia, and metabolic dysfunction [12]. They also possess variable activity at serotonin (5-HT₁A and 5-HT₂A) and other receptors, which may contribute to their anxiolytic and antidepressant properties [12]. Clinically, they tend to be well tolerated, though akathisia and insomnia remain notable side effects [12]. While long-term data are still being gathered, TGAs represent a promising step toward individualized treatment strategies that balance efficacy with improved metabolic and neurological safety.

**Table 1:** Classification and Key Clinical Characteristics of Antipsychotic Medications

Generation	Example Agents	Primary Receptor Targets	Major Clinical Effects	Key Adverse Effects	Relative Metabolic Risk
First-Generation (Typical)	Haloperidol, Fluphenazine, Chlorpromazine [12]	Dopamine D₂ antagonism [12]	Reduces positive symptoms [12]	EPS, tardive dyskinesia, NMS [12]	Low to moderate [12]
Second-Generation (Atypical)	Clozapine, Olanzapine, Risperidone, Quetiapine [12]	D₂ and 5-HT₂A antagonism; H₁, M₁, α₁ activity [12]	Improves positive and some negative symptoms [12]	Weight gain, dyslipidemia, insulin resistance [12]	High (esp. clozapine, olanzapine) [12]
Third-Generation (Partial Agonists)	Aripiprazole, Brexpiprazole, Cariprazine [12]	Partial D₂ & 5-HT₁A agonism; 5-HT₂A antagonism [12]	Mood stabilization, reduced EPS [12]	Akathisia, insomnia [12]	Low [12]

Cardiometabolic effects

The discussion of the growing use of antipsychotics for off-label purposes, particularly atypical agents, would be incomplete without addressing their potential cardiometabolic effects. These medications are well known to predispose users to dyslipidemia, weight gain, and diabetes, collectively heightening cardiovascular disease (CVD) risk [12]. Cardiometabolic disorders are themselves strongly associated with worsened psychiatric outcomes, including higher rates of depression, anxiety, cognitive decline, and reduced treatment adherence, even independent of medication effects [13-15]. In addition, weight-related stigma and resulting social withdrawal can further intensify this burden [16].

Patients with pre-existing metabolic disease are particularly vulnerable to rapid deterioration in cardiometabolic health when exposed to SGAs, a concern of growing magnitude given the large proportion of U.S. adults in this category [17].

CVD remains the leading cause of premature mortality among individuals with severe mental illness, shortening life expectancy by up to two decades [18]. While lifestyle and socioeconomic disparities contribute, antipsychotic therapy itself is a major modifiable driver of metabolic risk.

Clinical consequences and management implications

Once CVD develops, the relationship becomes bidirectional: depression after myocardial infarction, vascular cognitive impairment following stroke, and anxiety stemming from arrhythmias or heart failure exemplify how cardiovascular morbidity fuels psychiatric decline [19-21]. These psychiatric consequences, in turn, impair adherence to cardiometabolic treatment regimens, undermine lifestyle modifications, and increase the risk of recurrent cardiac events, creating a self-perpetuating feedback loop [22].

Antipsychotic therapy further compounds this cycle by contributing to cardiometabolic dysfunction, which heightens cardiovascular risk [12]. In turn, CVD exacerbates psychiatric morbidity, while worsening psychiatric symptoms undermine adherence and further elevate metabolic risk [23]. This interplay helps explain the disproportionately high mortality and disability observed among individuals with severe mental illness despite major therapeutic advances in psychiatry and cardiology [24].

Over the past two decades, extensive evidence has illuminated the public-health implications of antipsychotic-associated cardiometabolic disease. Rates of obesity, type 2 diabetes, and dyslipidemia among individuals receiving long-term antipsychotic therapy are consistently higher than in the general population, even after adjusting for baseline lifestyle factors [4]. Clozapine and olanzapine produce the greatest weight gain, sometimes exceeding 10 kg within a year, whereas ziprasidone and aripiprazole are tolerable on metabolic profiles [25]. Children and adolescents are especially vulnerable, often experiencing more rapid weight gain compared to adults. Untreated dyslipidemia subsequently increases the risk of coronary artery disease and stroke, compounding psychiatric morbidity [26-29].

Evidence also shows that antipsychotics can increase the risk of developing diabetes independent of weight gain and adiposity [30]. Thus, an assumption that DM associated with antipsychotics is because of obesogenic effects can be potentially an oversimplification [30-31]. Olanzapine and clozapine carry the highest risk due to their strong muscarinic M3 receptor-binding affinity; chronic M3 blockade disrupts pancreatic β-cell responsiveness and impairs glucose homeostasis [30]. Antipsychotics have been associated with a threefold increase in diabetes risk among children, an alarming finding given the rapid growth of off-label prescribing in this population [30]. Large cohort studies further indicate that individuals with schizophrenia or bipolar disorder have two- to three-fold higher rates of metabolic syndrome and a 50-70% greater risk of premature cardiovascular death [32-33].

Certain agents also prolong the QTc interval (e.g., ziprasidone, haloperidol), increasing the risk of sudden cardiac death [12]. Importantly, metabolic complications can emerge within months of treatment initiation yet often remain underrecognized in psychiatric settings [4]. In some reports, clinically significant weight gain appears within the first three months of therapy, yet fewer than half of patients receive recommended metabolic screening [34-35]. This lack of systematic monitoring delays detection of diabetes, hypertension, and dyslipidemia conditions that silently accelerate cardiovascular deterioration.

Beyond individual morbidity, the economic and societal impact is substantial [36]. The costs of managing diabetes, cardiovascular events, and related hospitalizations account for a major portion of healthcare expenditures associated with severe mental illness [36]. Indirect costs, including reduced work capacity, caregiver burden, and diminished quality of life, compound the issue, demonstrating that metabolic sequelae extend far beyond laboratory values [24].

Tables 2-3 summarize the relative metabolic liabilities of common antipsychotics and their comparative cardiometabolic risk based on receptor-binding profiles, while Table 4 illustrates the interplay between psychiatric illness and antipsychotic therapy that amplifies metabolic vulnerability.

**Table 2:** Relative Metabolic Liabilities of Common Antipsychotics +=low risk; ++=moderate; +++=high risk

Agent	Weight Gain	Dyslipidemia	Glucose Dysregulation	Overall Cardiometabolic Risk
Clozapine	+++ [5]	+++ [5]	+++ [5]	Very High [5]
Olanzapine	+++ [5]	++ [5]	+++ [5]	Very High [5]
Quetiapine	++ [5]	++ [5]	++ [5]	Moderate [5]
Risperidone	++ [5]	++ [5]	++ [5]	Moderate [5]
Paliperidone	++ [5]	+ [5]	++ [5]	Moderate [5]
Aripiprazole	+ [5]	+ [5]	+ [5]	Low [5]
Lurasidone	+ [5]	+ [5]	+ [5]	Low [5]
Ziprasidone	+ [5]	+ [5]	+ [5]	Low [5]

**Table 3:** Mechanistic Pathways Linking Antipsychotic Receptor Profiles to Metabolic Adverse Effects +=low risk; ++=moderate risk; +++=high risk

Primary Receptor Profile	Representative Agents	Weight Gain Risk	Glucose Dysregulation	Lipid Effects	Mechanistic Rationale
High H₁ + 5-HT₂C antagonism	Clozapine, Olanzapine [12]	+++ [5]	+++ [5]	+++ [5]	Drives appetite and reduces satiety; direct hypothalamic effects
Moderate 5-HT₂C + muscarinic antagonism	Quetiapine, Risperidone [12]	++ [5]	++ [5]	++ [5]	Partial serotonergic blockade; moderate insulin resistance
D₂ partial agonism, low histamine activity	Aripiprazole, Cariprazine [12]	+ [5]	+ [5]	+ [5]	Dopamine stabilization limits hyperphagia and insulin effects
High α₁, low H₁/M₃ activity	Ziprasidone, Lurasidone [12]	+ [5]	+ [5]	+ [5]	Minimal metabolic disruption; mild orthostasis only

**Table 4:** Biobehavioral Pathways Linking Serious Mental Illness and Second-Generation Antipsychotics to Metabolic Dysfunction This table summarizes major biological, endocrine, and behavioral mechanisms that contribute to cardiometabolic vulnerability in patients with serious mental illness and how second-generation antipsychotics (SGAs) further exacerbate these pathways. Baseline patient factors—including elevated pro-inflammatory cytokines (IL-6, TNF-α), hypothalamic–pituitary–adrenal (HPA) axis dysregulation, sedentary behavior, autonomic abnormalities, and sleep disruption—are shown alongside the corresponding SGA-related pharmacologic effects (e.g., histaminergic, muscarinic, and α₁-adrenergic blockade) that may synergistically worsen inflammation, insulin resistance, cardiovascular strain, and weight gain [25]. REM: Rapid Eye Movement

Risk Pathway	Baseline Patient Vulnerability	SGA-Related Exacerbation	Clinical Consequence
Inflammatory Cytokines (IL-6, TNF-α) [25]	↑ Baseline inflammation in schizophrenia, bipolar disorder [25]	SGAs amplify inflammation via weight gain and adiposity [25]	Exacerbated insulin resistance [25]
HPA Axis Dysregulation [25]	Chronic stress → cortisol excess [25]	SGAs increase leptin/cortisol levels [25]	Synergistic metabolic load [25]
Sedentary Behavior / Reduced Activity [25]	Cognitive deficits, amotivation [25]	Sedation (H₁, α₁ blockade) [25]	Reduced energy expenditure [25]
Autonomic Dysfunction [25]	HR variability loss in psychosis [25]	α₁ blockade → orthostasis, ↑ HR [25]	Cardiovascular strain [25]
Sleep Dysregulation [25]	Fragmented REM, circadian disruption [25]	SGAs alter melatonin and histamine tone [25]	Weight gain, metabolic risk [25]

Mechanisms of antipsychotic-induced cardiometabolic dysregulation

The cardiometabolic disturbances that result from antipsychotic interactions are from multiple receptor systems that regulate appetite, glucose metabolism, and lipid balance. Antagonism of histamine (H₁) and serotonin (5-HT₂C) receptors increases appetite and weight gain, while muscarinic (M₃) blockade impairs pancreatic β-cell insulin secretion [37-40]. Peripheral effects include decreased skeletal-muscle glucose uptake, hepatic insulin resistance, altered adipokine signaling, reduced adiponectin, and increased leptin resistance [37-40]. These contribute to insulin resistance, dyslipidemia, and central adiposity even before weight gain can occur. There is also an implication from studies that inflammatory and oxidative stress pathways, mitochondrial dysfunction, and hypothalamic AMPK dysregulation are also present, further increasing metabolic risk [37-40]. Ultimately, these receptor-mediated and downstream cellular effects explain the higher prevalence of metabolic syndrome and cardiovascular morbidity among antipsychotic users [37-40]. Beyond central appetite control, antipsychotics have peripheral effects on insulin and lipid metabolism. In skeletal muscle and hepatic tissue, they reduce insulin receptor signaling and GLUT4 translocation, leading to impaired glucose uptake and increased hepatic gluconeogenesis [37-40]. In adipose tissue, they promote adipogenesis, macrophage infiltration, and dysregulated adipokine secretion characterized by reduced adiponectin and increased leptin resistance, therefore amplifying systemic insulin resistance [37-40]. Chronic exposure also induces oxidative stress, mitochondrial dysfunction, and low-grade inflammation that further disrupt endothelial integrity and lipid handling [37-40]. Importantly, several studies show that these metabolic disturbances can occur independent of weight gain, implying receptor- and tissue-specific mechanisms intrinsic to drug exposure [37-40]. These pathways establish a multifactorial process of cardiometabolic dysregulation that bridges neurochemical, endocrine, and inflammatory domains, offering a biological explanation for the disproportionate burden of CVD observed among antipsychotic-treated populations [37-40].

Multiple professional organizations, including the American Diabetes Association (ADA), American Psychiatric Association (APA), and American Association of Clinical Endocrinology (AACE), recommend standardized baseline and follow-up metabolic monitoring for all patients prescribed antipsychotic therapy [4,35]. Despite this consensus, which dates back nearly two decades, compliance remains inconsistent in both psychiatric and primary-care settings, especially for vulnerable populations such as Medicaid patients and racial-ethnic minorities [6,34].

The prevailing hypothesis behind a lack of consistent follow-up metabolic monitoring is that it is most strongly associated with patient characteristics and health care utilization patterns, rather than prescriber specialty or knowledge [35]. Younger, healthier adults and those with less frequent contact with outpatient services are least likely to be screened, even though they may be at elevated risk due to antipsychotic exposure [35]. This is compounded by the fact that antipsychotic prescribing is distributed across diverse settings, including primary care and non-behavioral health providers, where responsibility for monitoring may be unclear, and coordination is often lacking [35]. System-level barriers such as unclear delineation of provider roles, fragmented care between psychiatry and primary care, and lack of integrated health records further impede consistent monitoring [35].

Suggested strategies for managing cardiometabolic consequences of antipsychotics

Baseline Evaluation

Before initiating therapy, clinicians should document weight and BMI, waist circumference, fasting glucose or A1C, lipid profile, blood pressure, and, when indicated, an electrocardiogram (ECG) to assess QTc risk. Baseline data allow early identification of at-risk individuals, particularly those with pre-existing obesity, diabetes, or dyslipidemia.

Follow-up Monitoring

Reassessment is recommended at 4, 8, and 12 weeks, again at 6 months, and annually thereafter, with shorter intervals for patients exhibiting rapid weight gain or abnormal laboratory findings. Weight gain exceeding 7% of baseline or new-onset glucose intolerance should prompt therapeutic reevaluation [4]. However, real-world data reveal that fewer than half of patients receive lipid or glucose screening after initiating antipsychotics, even in integrated health systems [35]. Reported barriers include lack of coordination between psychiatry and primary care, unclear provider responsibility, fragmented EHR systems, and insufficient reimbursement for preventive laboratory testing [6,35]. Addressing these systemic gaps is essential to translating guideline recommendations into meaningful patient outcomes.

Additional management and mitigation strategies

Given the high prevalence of metabolic complications, comprehensive management must combine behavioral, pharmacologic, and collaborative approaches tailored to individual risk profiles.

Lifestyle Interventions

Lifestyle modification remains the cornerstone of prevention. Psychoeducation emphasizing balanced nutrition, portion control, regular physical activity, and reduction of sedentary behavior has demonstrated moderate efficacy in reducing weight gain and improving glycemic parameters among patients on SGAs [24]. Behavioral counseling and structured exercise programs should ideally begin before treatment initiation to establish sustainable habits.

Pharmacologic Adjuncts

When lifestyle measures are insufficient, metformin, supported by multiple randomized controlled trials, can attenuate weight gain and insulin resistance, particularly with clozapine and olanzapine [4]. More recently, GLP-1 receptor agonists such as liraglutide and semaglutide have shown clinically meaningful reductions in body weight and A1C among antipsychotic-treated populations, though long-term psychiatric safety data remain limited. These agents may become important adjuncts for high-risk patients intolerant of metformin or with established metabolic syndrome.

Switching Strategies

Transitioning from high-risk agents (e.g., clozapine, olanzapine) to lower-risk alternatives (e.g., aripiprazole, ziprasidone, lurasidone) can yield significant metabolic improvements without compromising psychiatric stability in many cases [4, 30-31]. Nonetheless, switching should be done cautiously with close symptom monitoring to prevent relapse.

Collaborative and Integrated Care

Optimal outcomes require multidisciplinary coordination between psychiatry, primary care, and endocrinology. Shared-care models-where psychiatrists initiate monitoring and primary-care providers maintain ongoing surveillance, improve adherence to screening protocols, and facilitate timely intervention for emerging abnormalities [6,18]. Embedding clinical pharmacists or nurse practitioners into mental-health teams further enhances medication review and patient education.

Policy Interventions

A majority of interventions to reduce off-label antipsychotic usage can be argued to be only accomplished at the policy level. Strategies such as prior authorization requirements, quality monitoring programs, and targeted oversight have demonstrated great effectiveness in reducing off-label antipsychotic use, particularly in vulnerable populations like the youth and in older adults [41-46]. For example, Medicaid policy initiatives, including prior authorization and quality improvements, were associated with substantial declines in antipsychotic prescribing among targeted groups [47]. Federal oversight and CMS attention have focused largely on curbing inappropriate use, especially in older adults, where the risks are well documented [48-49]. Prior authorization programs are among the most strongly supported interventions to reduce inappropriate off-label antipsychotic usage [41-43].

A mandatory peer-review PA program in Washington State resulted in a 38% decline in antipsychotic use among Medicaid-insured children within two years, compared to no change in controls [43]. Similarly, a systematic review found that more than half of PA interventions were associated with significant reductions in antipsychotic prescribing or improved adherence to best practices [41].PA policies also have “spillover” effects, reducing prescribing in commercially insured youth in states where Medicaid PA was implemented [50]. However, opponents, on the other hand, have argued that off-label antipsychotic usage still remained common despite these implementations, suggesting that policy-level changes are not enough alone to necessarily solve this issue of overusing antipsychotic drugs [46,51].

Education and prescriber behavior change

It is absolutely crucial to implement intensive education to update clinicians on the risks and limited evidence for off-label antipsychotic use to enhance prescribing quality [2,51,52]. Behavioral economic interventions such as peer comparison letters have been shown to shift high-volume prescribers toward evidence-based practice [53,54]. As mentioned previously in this literature review and additionally, supported by expert consensus and systemic reviews, there is a demonstration that most off-label antipsychotic use is not supported by strong evidence and is often inappropriate [2]. This discovery underscores an educational deficiency and a need for ongoing feedback.

System-level changes

There must be an improved integration between primary care and psychiatry; persistent competency gaps still exist. General practitioners often lack confidence and training to manage antipsychotic medications, while psychiatrists may not address cardiometabolic risks associated with antipsychotics [55,56]. Psychiatry as a profession itself would benefit from additional medical training and additional system-level support because psychiatrists are affected by fragmented care systems and inadequate postgraduate training [57-58,59-61]. Both qualitative and survey-based studies show psychiatrists often lack training in the management of common cardiometabolic comorbidities [57,58].

For example, a cross-sectional survey found that while psychiatry residents encounter patients with these conditions, they scored much lower than family medicine residents on knowledge assessments for their management, and over 60% of psychiatry residents felt their training in these areas was inadequate [57]. There is also a demand for better access to non-pharmacological interventions and enhanced communication. This will be crucial to address gaps that can lead to inappropriate long-term antipsychotic use [55]. Fragmented care, lack of psychiatric follow-up, and limited availability of psychological therapies contribute to overprescribing and make deprescribing more difficult. Addressing these barriers requires organizational and contractual reforms, as well as investment in alternative treatments [55,56]. Another unaddressed issue is to tackle clinician fears of medicolegal consequences and to provide clear, evidence-based guidelines for deprescribing to encourage more appropriate use of antipsychotics [55,62,63]. Monitoring and feedback systems, such as regular audits and public reporting, can reinforce best practices and accountability [55,56].

More possible future directions

Personalized Psychiatry and Genomic Risk Profiling: Emerging research suggests that genetic polymorphisms affecting dopamine, serotonin, and leptin pathways may predict susceptibility to antipsychotic-induced weight gain and insulin resistance. Incorporating pharmacogenomic screening into prescribing practices could allow pre-emptive identification of high-risk individuals and enable personalized drug selection in the near future.

Development of Metabolically Neutral Antipsychotics

Ongoing psychopharmacology research aims to design agents that retain antipsychotic efficacy while minimizing metabolic liability. TGAs such as aripiprazole, brexpiprazole, and cariprazine already represent partial progress, and future compounds targeting selective D₂/D₃ modulation or biased agonism at 5-HT₂A receptors may further reduce cardiometabolic risk.

Integrated Care Models

The future of psychiatric practice will increasingly hinge on integration with cardiology and endocrinology services. Collaborative clinics combining metabolic screening, medication management, and lifestyle counseling under one setting have demonstrated improved outcomes and reduced hospitalizations among individuals with severe mental illness [18,36]. Expanding such models within community mental-health systems represents a crucial step toward bridging the gap between psychiatric and physical healthcare.

**Figure 2:**
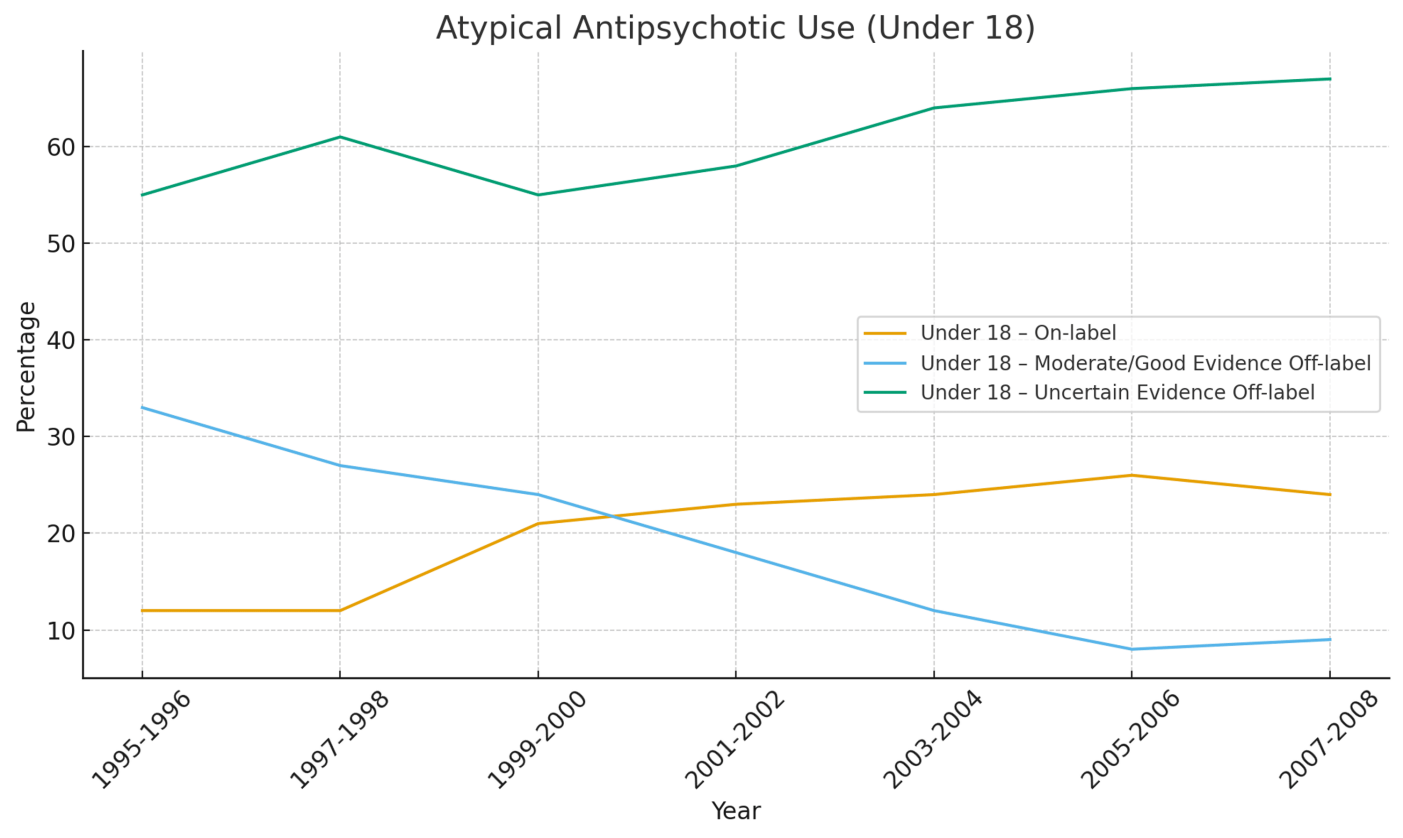
Atypical Antipsychotic Use Under 18 What the numbers show is uncertain-evidence off-label use rises from 55% → 67%, in-label use rises (12% → 24%), but still is small and moderate/good-evidence off-label use collapses from 33% → 9% [2]. Children became increasingly exposed to atypical antipsychotics for conditions with little to no supporting evidence. This aligns with the expansion of antipsychotic use for ADHD, aggression, mood dysregulation, very limited pediatric FDA indications at that time, and concerns about metabolic risks in youth. Clinically: this is the population at highest risk for long-term weight gain and cardiometabolic disease, yet the evidence base was weakest [2].

**Figure 3:**
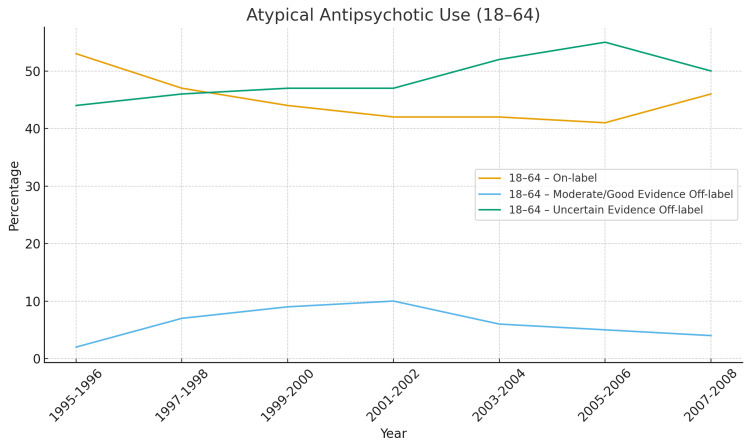
Atypical Antipsychotic Use 18-64 Of the adults presented here, a majority were given antipsychotics off-label. What the numbers show is that on-label use decreases then stabilizes (53% → 41–46%), off-label uncertain evidence stays high (44–55%), and even in adults, the group with the widest FDA approvals off-label prescribing remained higher than on-label throughout the entire 13-year span [2].

**Figure 4:**
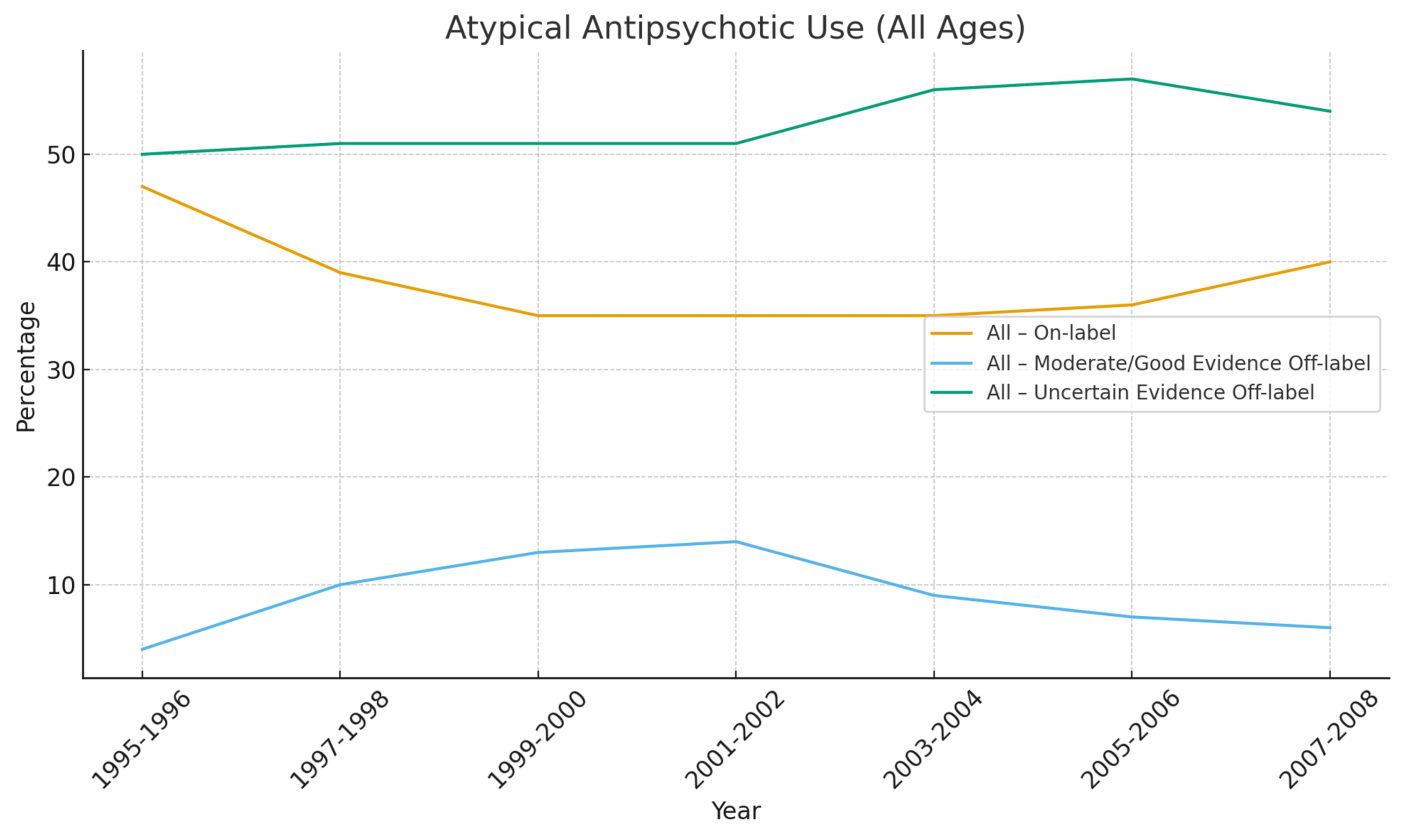
Antitypical Antipsychotic Use in All Ages Uncertain-evidence off-label use consistently dominated antipsychotic prescribing (57–80%) while on-label use was lower and moderate/good-evidence off-label use only briefly increased before declining again indicating that older adults were the most aggressively treated with antipsychotics for the weakest-evidence indications, particularly dementia-related agitation, delirium, and behavioral symptoms in long-term care settings, all of which carry high mortality risk and black-box warnings [2].

**Figure 5:**
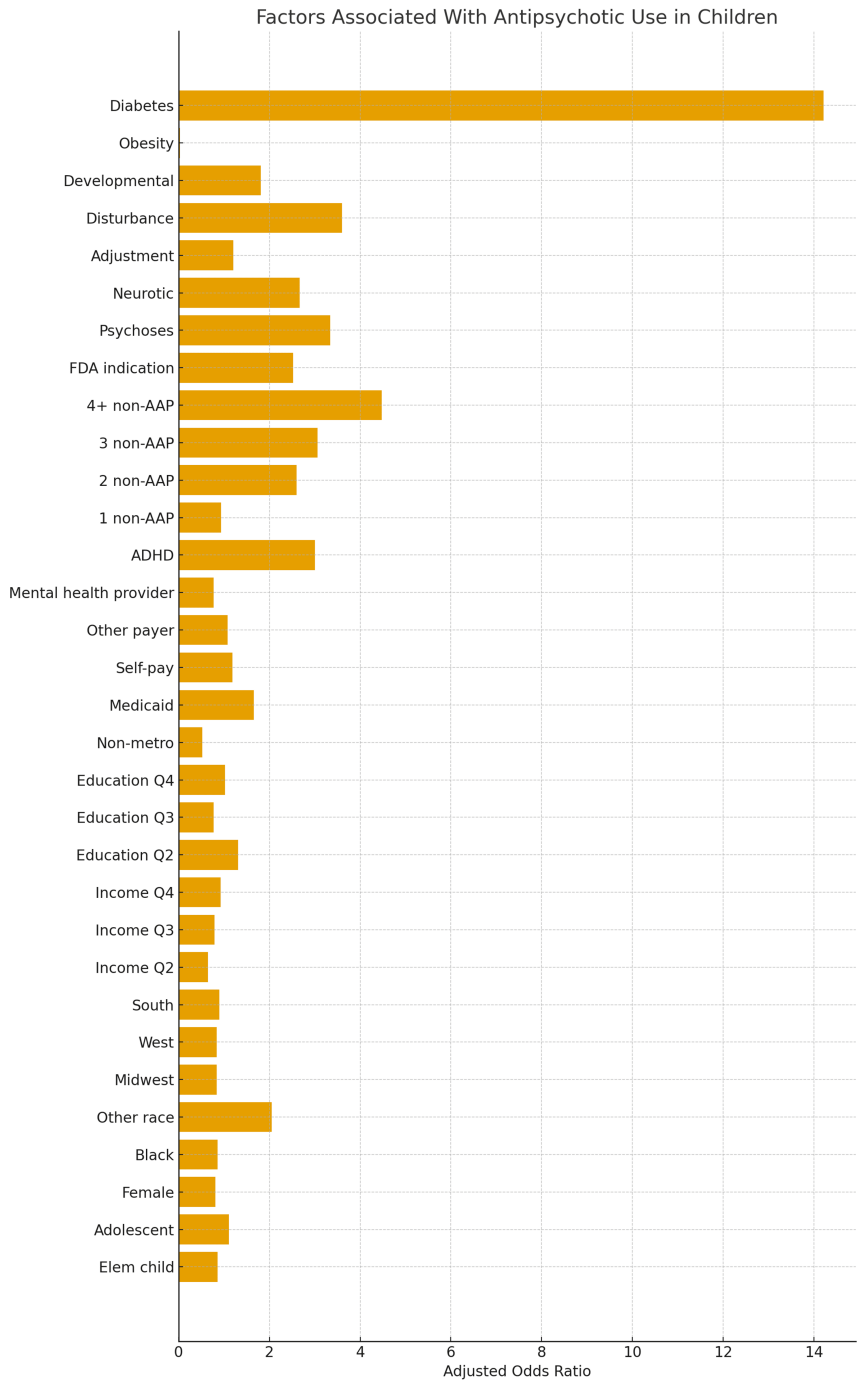
Factors Associated with Atypical Antipsychotic Use in Children An odds ratio greater than 1 means the factor is associated with a higher likelihood of receiving an antipsychotic, while an odds ratio less than 1 would indicate a lower likelihood. The further the bar extends to the right, the stronger the association with antipsychotics [64]. Some of the categories the y-axis means [64]: Developmental, Disturbance, Adjustment, and Neurotic Disorders; broad psychiatric diagnostic groups; Developmental disorders include autism spectrum disorder and intellectual disability. Disturbance includes conduct disorders, behavioral dysregulation, or oppositional problems; Adjustment refers to emotional or behavioral reactions to stressors. Neurotic disorders include anxiety disorders. Psychoses: This refers to diagnoses such as schizophrenia or other primary psychotic disorders. FDA indication: This means the child has a diagnosis for which antipsychotics are officially FDA-approved “1 non-AAP,” “2 non-AAP,” “3 non-AAP,” “4+ non-AAP”: Refer to the number of antipsychotic psychotropic medications the child is already taking. The higher the number, the more likely a clinician is to add an antipsychotic. “4+ non-AAP” represents very high polypharmacy and strongly increases antipsychotic use Insurance/Payer Factors (Medicaid, Self-pay, Other payer): Mental health provider: Access to psychiatric care modestly increases the chance of antipsychotic prescribing. Sociodemographic Factors (Income quartiles, Education quartiles, Metro vs non-metro, Region, Race, Sex): These measure the influence of socioeconomic background and demographics. Income and education quartiles compare families by neighborhood socioeconomic status. South, Midwest, and West refer to U.S. regional prescribing differences. Black, Other race, and female reflect demographic variation in treatment patterns. Age groups (Elementary child, Adolescent) [64].

**Figure 6:**
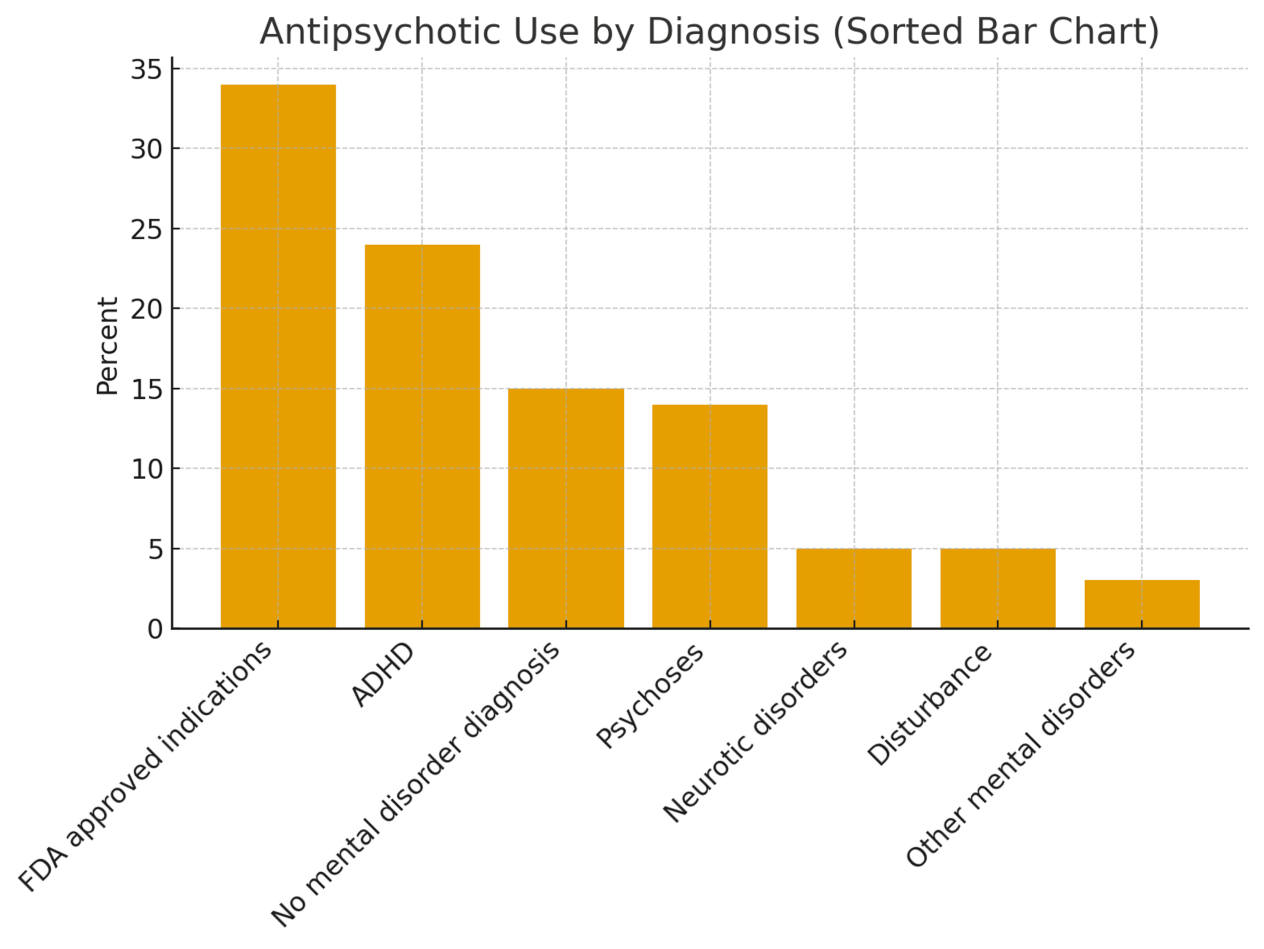
Mental Health Conditions Related To Antipsychotic Prescriptions for Children This graph displays the distribution of antipsychotic prescriptions in children according to diagnostic category, arranged from most to least common. FDA-approved indications account for the largest share at 34%, reflecting conditions such as autism-related irritability, Tourette’s disorder, and schizophrenia or bipolar disorder in adolescents—showing that a substantial portion of prescribing is on-label and appropriate. The second most common category is ADHD at 24%, despite antipsychotics not being FDA-approved for this condition; this highlights significant off-label use, often aimed at managing severe impulsivity, aggression, or behavioral dysregulation. Notably, 15% of prescriptions were given to children without any recorded mental health diagnosis, suggesting that antipsychotics may be used for behavioral control, nonspecific agitation, medical issues, or possibly undocumented psychiatric conditions, an observation widely viewed as a prescribing red flag. Psychotic disorders make up only 14% of use, meaning that most pediatric antipsychotic prescribing is not for primary psychosis. The remaining categories—disturbance disorders (5%), neurotic disorders (5%), and other diagnoses (3%) account for small portions of prescribing and likely represent anxiety, conduct-related conditions, disruptive mood dysregulation, and similar presentations. Overall, the graph underscores that the majority of antipsychotic prescribing in children occurs for off-label reasons such as ADHD or aggression, or even without a documented diagnosis, a major and ongoing concern in pediatric psychopharmacology [64].

Figures 2-6 below have been produced using pre-existing data collected regarding antipsychotic trends to help readers with ease of further interpreting trends regarding antipsychotics [64,2].

## Conclusions

Antipsychotic medications remain a cornerstone of psychiatric treatment, but their widespread use carries a significant responsibility to monitor and manage cardiometabolic risks. Effective care extends beyond symptom control; it must also address the long-term physical health consequences that accompany these therapies. Consistent metabolic screening, early lifestyle intervention, and collaborative coordination between psychiatry and primary care are essential to prevent avoidable complications. Future efforts should focus on developing safer, metabolically neutral agents and integrating personalized approaches that balance psychiatric stability with overall well-being. Protecting the cardiovascular and metabolic health of patients with mental illness is not only a clinical priority but also a fundamental aspect of compassionate, comprehensive care.
